# Epigenetic Changes in Response to Tai Chi Practice: A Pilot Investigation of DNA Methylation Marks

**DOI:** 10.1155/2012/841810

**Published:** 2012-06-05

**Authors:** Hua Ren, Veronica Collins, Sandy J. Clarke, Jin-Song Han, Paul Lam, Fiona Clay, Lara M. Williamson, K. H. Andy Choo

**Affiliations:** ^1^Chromosome Research, Murdoch Childrens Research Institute, Royal Children's Hospital, Parkville, VIC 3052, Australia; ^2^Public Health Genetics, Murdoch Childrens Research Institute, Royal Children's Hospital, Parkville, VIC 3052, Australia; ^3^Statistical Consulting Centre, University of Melbourne, Parkville, VIC 3010, Australia; ^4^Tai Chi Australia, VIC 3146, Australia; ^5^Tai Chi for Health Institute, Narwee, NSW 2209, Australia; ^6^Department of Paediatrics, University of Melbourne, Parkville, VIC 3052, Australia

## Abstract

Tai chi exercise has been shown to improve physiological and psychosocial functions, well-being, quality of life, and disease conditions. The biological mechanisms by which tai chi exerts its holistic effects remain unknown. We investigated whether tai chi practice results in positive epigenetic changes at the molecular level. *Design*. The DNA methylation profiles of sixty CpG-dinucleotide marks in female tai chi practitioners (*N* = 237; 45–88 years old) who have been practising tai chi for three or more years were compared with those of age-matched control females (*N* = 263) who have never practised tai chi. *Results*. Six CpG marks originating from three different chromosomes reveal a significant difference (*P* < 0.05) between the two cohorts. Four marks show losses while two marks show gains in DNA methylation with age in the controls. In the tai chi cohort all six marks demonstrate significant slowing (by 5–70%) of the age-related methylation losses or gains observed in the controls, suggesting that tai chi practice may be associated with measurable beneficial epigenetic changes. *Conclusions*. The results implicate the potential use of DNA methylation as an epigenetic biomarker to better understand the biological mechanisms and the health and therapeutic efficacies of tai chi.

## 1. Introduction

Tai chi is a traditional Chinese health practice with an uninterrupted history of development dating back thousands of years. Nowadays tai chi is practised as a mind-body exercise by millions all over the world for therapeutic purposes and to improve health. During tai chi, the practitioner performs a series of specially designed movements slowly, gently, and in combination with deep breathing, relaxation techniques, and a tranquil or meditative state of mind. When practised correctly, tai chi is thought to strengthen the body's vital energy (or *qi*) and enhance the passage of this energy throughout the body to confer its health-promoting effects.

An increasing number of studies have pointed to physiological and psychosocial benefits of tai chi in terms of improving quality of life and physical and physiological function including musculoskeletal strength, flexibility, cardiovascular fitness, and immune response [1–3]. Other studies have shown that tai chi offers therapeutic benefits for patients with chronic and debilitating conditions such as fibromyalgia [4, 5], Parkinson's disease [6], rheumatoid arthritis, and osteoarthritis [7, 8]. However, the biological mechanisms by which tai chi exerts its effects remain unknown, and there are no known biomarkers that can be used to determine the efficacy of tai chi or changes that may accompany tai chi practice at the molecular or cellular level.

Epigenetics is the study of heritable mitotic or meiotic changes in the function of the genome without directly altering its DNA sequence. Through epigenetic modifications, cells respond to different internal or external stimuli to achieve changes in gene expression and gene function. Studies have shown that DNA methylation profiles, one of the epigenetic marks, vary within an individual in different tissues and that DNA methylation functions decline with age [9–13] and can be modified by exercise [14–16] and environmental stimuli such as exposure to sun, asbestos, arsenic, alcohol, and tobacco [11, 17–20]. Studies have also indicated that changes in epigenetic marks may play a significant role in the aetiology of common human diseases such as cancer, diabetes, immune disorders, and autism [21–23], and that a decline in epigenetic functions provides a mechanism for the late onset of some common diseases including cancer [9, 24].

Tai chi is a mind-body exercise that has been reported to demonstrate a holistic positive effect on a significant number of physical, physiological, and psychosocial functions [1, 5, 8, 25–27]. We postulate that the beneficial effects of tai chi exercise are associated with subcellular changes that have an epigenetic origin. To this end, we investigate here the effects of tai chi on changes in the pattern of DNA methylation at specific CpG sites within the genome. Specifically, we compared the DNA methylation profiles of 60 CpG sites originating from different chromosomes between a cohort of tai chi practitioners and a matched control cohort of people who have never practiced tai chi.

## 2. Materials and Methods

### 2.1. Study Participants

Tai chi practitioners were recruited from the tai chi schools across the states of Victoria and Tasmania in Australia. At the time of recruitment, the participants had been practising Yang-style tai chi for 3 years or more for at least 1 hour per week. Due to a predominance of women who were in their midforties and older in these schools, to control for possible age and sex effects, we restricted our study to female participants who were 45 years or older. In all, 237 female tai chi practitioners within the age range of 45–88 years were recruited. As controls, participants who had never practised tai chi were recruited from the local communities, and some who had just enrolled in beginners' tai chi classes. A total of 263 age- and ethnicity-matched control individuals were included. All participants completed a detailed questionnaire to provide information regarding demographics, details about tai chi practice and other exercise, smoking, health conditions, height and weight, and anxiety and perceived stress. Situational anxiety was measured using the state subscale (short form) of the Spielberger State-Trait Anxiety Inventory [28]. Perceived stress was measured using the Perceived Stress Scale (4-item form) [29]—a commonly used measure of general perceived stress in studies of both mental and physical health. The research protocol was approved for human study by the Human Ethics Committee of The Royal Children's Hospital, Melbourne, Australia. All participants signed informed consent prior to participating in the study.

### 2.2. Mouthwash Collection and DNA Isolation

The participants rinsed their mouths 15 times with 10 mL of Cool Mint Listerine mouthwash solution purchased from a local supermarket. The mouthwash was then collected in 50 mL falcon tube, and DNA was isolated using Puregene DNA purification kit (Gentra, USA) following the manufacturer's instructions and stored at −20°C.

### 2.3. Bisulfite Conversion

1 *μ*g of genomic DNA was converted by bisulfite treatment using EZ-96 DNA Methylation-Gold Kit from Zymo Research Corporation (Orange, CA) following the instructions of the manufacturer. The final volume of the bisulfite-converted DNA was 15 *μ*L. 2 *μ*L of the converted DNA were used for the methylation analysis of each of the genetic loci.

### 2.4. Genetic Loci and Primer Design

The primers (Table 3) were designed with the assistance of MethPrimer (http://www.urogene.org/methprimer/) in either the subtelomere regions of chromosomes or the promoter regions of selected genes. The primers and amplicons were further checked for CpGs, T fragmentation, spectra, and report using R console 2.9.2 (The R Foundation for Statistical Computing, 2009).

### 2.5. DNA Methylation Analysis

DNA methylation analysis was performed using the Sequenom EpiTYPER Platform Matrix-Assisted Laser Desorption/Ionization Time-of-Flight Mass Spectrometry (MALDI-TOF MS) [30]. All reagents, equipments, and software were from The SEQUENOM, Inc., San Diego. Briefly, 2 *μ*L of bisulfite-treated DNA was amplified in 10 *μ*L final reaction volume using HotStar Taq DNA Polymerase (Qiagen GmbH, Germany) (95°C/15 min; 45 cycles: 95°C/20 sec, 55°C/30 sec, and 72°C/30 sec; then 72°C/4 min). 5 *μ*L of the PCR product were then neutralised using shrimp alkaline phosphatase in a 7 *μ*L reaction (37°C/20 min, 85°C/5 min) to remove remained dNTPs. The RNA transcription and T-cleavage reaction was carried out at 37°C for 3 hrs using MassCLEAVE T7 kit (The SEQUENOM, Inc., San Diego) in a 7-*μ*L reaction containing 2 *μ*L of SAP reaction product, 0.89 *μ*L of 5x T7 polymerase buffer, 0.22 *μ*L of T-cleavage mix, 0.22 *μ*L of DTT (100 mM), 0.4 *μ*L of T7 RNA and DNA polymerase, 0.06 *μ*L of RNase A to give a final concentration of 0.09 mg/mL, and 3.21 *μ*L of RNase-free ddH_2_O. The fragmented RNA molecules were conditioned using 6 mg of MassArray Clean Resin to remove free ions in the reaction before spotted onto spectroCHIPs using the Nanodispenser RS 1000 and then processed for methylation analysis using the MALDI-TOF MS. Data were viewed and further analysed at an uncertainty threshold of 0.1 on EpiTYPER software 1.0.

### 2.6. Statistical Analysis

The methylation data were analysed using the SPSS software (PASW Statistics 18, http://www.spss.com/). As the response variables are proportions, they were often concentrated at the boundaries of 0 and 1 and therefore did not satisfy the assumptions of a general linear model. To correct for this, an angular transformation was used [31]. An assessment of the residuals of the models used indicated that this correction was successful for the majority of markers and that the general linear model assumptions were satisfied.

We analysed the impact of a variety of potential confounders that may be associated with DNA methylation. These variables were (1) BMI (Body Mass Index) in four categories (underweight, normal, overweight and obese); (2) smoking status in three categories (never smoked, smoked in the past, and currently smoke); (3) medical history of type 2 diabetes (yes or no); (4) medical history of cardiovascular disease (yes or no); (5) medical history of hypertension (yes or no); (6) age as a continuous variable. As there was interest in determining how the impact of tai chi on DNA methylation depends on age, the interaction between these two variables was also included.

## 3. Results

### 3.1. Characteristics of the Participants

As summarised in Table 1, the main characteristics of the participants were not significantly different between the tai chi and control cohorts. A similar proportion of participants in the tai chi and control group (*P* = 0.99) performed regular exercise other than tai chi. These exercises included running, cycling, gymnasium workout, aerobics, circuit classes, walking, pilates, yoga, or meditation. As tai chi is a mind-body exercise, we noted the number of participants who were doing other mind-body exercises such as yoga and meditation and found only a small and approximately similar percentage (*P* = 0.37) practising these, suggesting that they are unlikely to interfere with the outcomes of our study.

Hypertension condition showed a higher percentage among the tai chi practitioners compared to the controls (*P* = 0.06). This could be explained by a substantial proportion (26%; Table 2) of the tai chi participants reporting that they took up tai chi to help with specific health problems, suggesting a possible bias towards poorer health conditions of the tai chi cohort at the outset.

There were slightly more tai chi participants (*P* = 0.04) describing themselves as feeling calm, relaxed or content, and less tense, upset, or worried, at the time when the survey was done (Table 1; situational anxiety).

Among the 237 tai chi practitioners recruited, tai chi history ranged from 3 to over 40 years, and the time of tai chi practice varied from 1 to 10 hour per week. Ideally we would have analysed the data according to practice hours and years but the participant numbers in each group were too small to give statistically reliable results.

### 3.2. DNA Methylation Studies

We investigated the DNA methylation properties of sixty CpGs on seven genetic loci originating from six different chromosomes (Table 3). These loci were chosen because earlier studies have implicated their association with age-related DNA methylation changes [13, 18, 32, 33]. The 17p and Xp13 loci were from the p-arm subtelomere regions of chromosomes 17 and X, while the Rad50, Esr1, WRN, Ercc1, and G6PD loci were from the promoter regions of the corresponding genes on chromosomes 5, 6, 8, 19, and X, respectively.

#### 3.2.1. DNA Methylation, BMI, Smoking, and Alcohol Intake

We observed no significant association between BMI level, smoking status, or amount of alcohol consumed and percentage methylation for all sixty CpGs (data not shown) in line with other studies [14, 15].

#### 3.2.2. DNA Methylation and Tai Chi

Of the sixty CpGs, six (17p_7, Xp13_1, Rad50_2, Rad50_10, G6PD_6, and G6PD_7) were found to show a significant difference (*P* < 0.05) in DNA methylation between the tai chi and control cohorts (Table 4).

**Figure 1:**
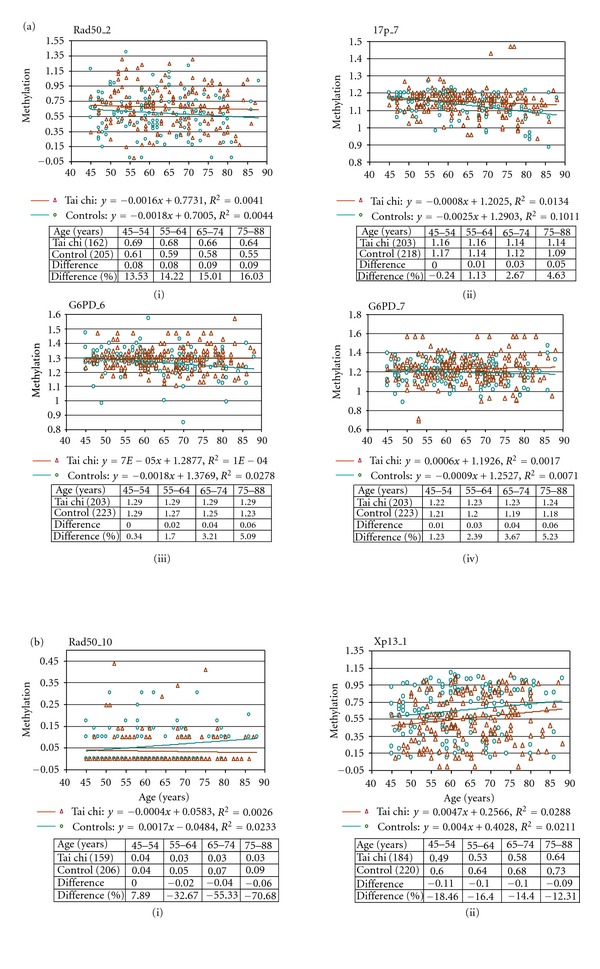
Comparison of DNA methylation profiles against age for CpG dinucleotide marks that show a significant difference between the tai chi and control cohorts. ((a), i–iv) CpG marks Rad50_2, 17p_7, G6PD_6, and G6PD_7, showing normal DNA methylation losses in the control cohort (green) across age. Slower loss rates are seen for these marks in the tai chi cohort (red), by up to 16.03%, 4.63%, 5.09%, and 5.23%, respectively, compared to those of the control cohort, as shown by the tables below the graphs. ((b), i-ii) CpG marks Rad50_10 and Xp13_1, showing normal age-related DNA methylation gains in the control cohort (green). Slower gain rates are seen for these marks in the tai chi cohort (red), by up to 70.68% and 18.46%, respectively, compared to those of the control cohort, as shown by the tables below the graphs. Values in the tables represent average DNA methylation values for the age categories indicated, based on the linear model represented by the fitted line. “% difference” denotes the difference between the tai chi and control values expressed as a percentage of the control value. As methylation results were not available for all the participants for the individual CpG marks, the numbers of participants included in these analyses were less than the total and are shown in brackets.

Rad50_2, 17p_7, G6PD_6, and G6PD_7 showed a decreasing DNA methylation trend across age in the control cohort (Figure 1(a), i–iv), suggesting the progressive loss of DNA methylation on these CpGs with age in the normal population. For these four marks, the tai chi practitioners as a group showed DNA methylation levels that were consistently lower than those of the controls across age (Figure 1(a), i–iv). Rad50_2 appeared to respond to the positive effects of tai chi across the 45–88-year-old age range studied (by approximately 13.53–16.03% across the different age categories compared to the controls, as indicated in the table in Figure 1(a)-i). In contrast, for the 17p_7, G6PD_6, and G6PD_7, the positive effects of tai chi became apparent only around or after 55 years of age, reaching a maximum difference of approximately 4.63%, 5.09%, and 5.23%, respectively, after the age of 75 years compared to the controls (see the tables in Figure 1(a), ii–iv).

Unlike the decreasing normal DNA methylation trends seen in the Rad50_2, 17p_7, G6PD_6, and G6PD_7 marks, age-related increasing normal DNA methylation trends were observed for the Rad50_10 and Xp13_1 in the controls (Figure 1(b), i-ii). These results suggested a progressive gain of DNA methylation on the Rad50_10 and Xp13_1 with age in the normal population. For these two marks, the tai chi practitioners showed DNA methylation trends that were indicative of the slowing of the age-related gains seen in the controls. As with Rad50_2, the Xp13_1 mark seemed to respond to the positive effects of tai chi across the 45–88-year-old age range studied (by approximately 12.31–18.46% across the different age categories compared to the controls, as shown in the table in Figure 1(b)-ii). In contrast, the Rad50_10 appeared to behave more like the 17p_7, G6PD_6, and G6PD_7 in that the positive effects of tai chi did not become apparent until after 55 years of age, reaching a maximum difference of approximately 70.68% after the age of 75 years compared to the controls (see the table in Figure 1(b)-i).

## 4. Discussion

DNA methylation provides an essential epigenetic mechanism that influences a wide range of biological functions including transcription activity, chromatin organisation, imprinting, cellular differentiation, and chromosome stability. Previous studies have established that the patterns of DNA methylation at specific genetic loci display tissue [12, 18, 34–37] and gender differences [13, 38–43] and can be modified by environmental influences such as diet [44–48], famine [49], or exposure to sun, tobacco, alcohol, arsenic, and asbestos [11, 17–20]. In animal studies, maternal behaviour involving high levels of maternal licking and grooming of pups is associated with changes in CpG methylation in the pups [50]. It has also been reported that DNA methylation profiles can change with age [9–13, 18, 19, 38, 40].

In this study, we have investigated whether tai chi practice is associated with changes in DNA methylation and, if so, whether the changes are beneficial (as defined by the slowing of age-related changes). To address these questions, we have determined and compared the methylation profiles of 60 CpGs from six different chromosomes between female tai chi practitioners and the matched controls. We have identified six CpG marks originating from chromosomes 5, 17, and X that show a significant difference (*P* = 0.001–0.031) between the two cohorts. Four of these CpGs show losses in DNA methylation while the remaining two demonstrate gains in DNA methylation with age in the controls. The results indicate that the two adjacently located CpGs (G6PD_6 and G6PD_7, 20 bp apart; see Table 4) within the promoter region of the G6PD gene both give decreasing normal DNA methylation trends with age. In contrast, within the Rad50 gene promoter region, while a decreasing normal methylation trend is seen for the Rad50_2, an increasing trend is seen for the Rad50_10 situated 147 bp downstream (Table 4). Previous studies have described variability in DNA methylation of distinct CpG marks that are situated in very close proximity [51, 52].

Notably, for all six CpGs, we have observed a slowing of the age-related trends for DNA methylation losses or gains in the tai chi cohort compared to the control cohort. The maximum amount of slowing ranged from approximately 5% to 70% for the different marks, with the Rad50_10 mark from the promoter region of the DNA double-strand break repair protein gene Rad50 [53] showing the greatest effect of slowing. There also appear to be two noticeable timing patterns with respect to when a CpG shows a response to the effects of tai chi. For Rad50_2 and Xp13_1, the effects of tai chi are detectable from (and presumably before) 45 years of age and thereafter across the 45–88 age range. For the remaining four marks, significant methylation differences between the two cohorts do not become apparent until after 50–55 years of age. One possible explanation for this observation is that tai chi has no effect on the methylation profiles of these four CpGs until after this age. An alternative and tantalising explanation is that a significant deterioration of the normal DNA methylation profiles for these four CpGs does not occur until after the postmenopausal age of 50–55 years and that tai chi is effective in slowing this deterioration when it occurs.

Other studies have described nonuniform changes of DNA methylation in different CpGs over time [9, 18]. The changes can result in, or increase the risk of, disease such as Beckwith-Wiedemann, Rett, and ICF (Immunodeficiency, Centromere instability and Facial anomalies) syndromes and cancers [54–61]. Age-related decline of DNA methylation reflects the gradual deterioration of important epigenetic regulatory functions and genetic control, providing a mechanism for late onset of common diseases including cancer [9, 24]. Our observed slower cross-sectional decline in the DNA methylation of all six CpGs in the tai chi cohort compared to those of the controls suggests that tai chi practice has a beneficial effect in protecting against the decay of epigenetic functions with age.

How tai chi brings about such a protection is unclear. Numerous studies have pointed to a positive effect of tai chi on a significant number of physical, physiological, and psychosocial functions including balance control, flexibility, cardiovascular fitness, falls in the elderly, musculoskeletal conditions such as osteoarthritis and rheumatoid arthritis, hypertension, bone mineral density, neuromuscular function, the cardiovascular, respiratory, endocrine, and immune systems, psychological responses including depression and perceived health status, and quality of life including activity tolerance, pain management, kinaesthetic sense, sleep quality, and stress reduction [1, 5, 7, 8, 25–27]. Studies have also shown the benefit of tai chi on bone mineral density and neuromuscular function in postmenopausal women [25, 62]. Where tai chi was compared to other forms of exercise, such as stretching and/or resistance-training exercises used in the fibromyalgia and Parkinson's Disease studies [5, 6], significantly better health outcomes were reported for tai chi; however, a systematic comparison of tai chi and other forms of exercise has not been done. Our study was not designed to answer the question of whether general exercise is related to epigenetic changes. We collected data on exercise to adjust for possible confounding. As Table 1 indicates, a similar proportion of tai chi and control participants (86% and 85%, resp.; *P* = 0.99) regularly performed non-tai chi exercise. There was a range of non-tai chi exercise being done by participants and we do not have enough details about types and intensities of exercise to further analyse whether general exercise is related to epigenetic changes. Our hypothesis related specifically to mind-body exercise, that is, tai chi.

As a complex intervention tool, tai chi is thought to impart its beneficial effects through the integrated improvement of the well-being of the individual at the physical, psychosocial, physiological, emotional, behavioural, and spiritual levels. As the results of this preliminary study suggest, such a holistic intervention approach may, at a more fundamental level, impact directly on the epigenetic and molecular wellbeing of the cells.

This study has a number of limitations. As we performed simultaneous statistical testing on multiple CpGs, the chance of false positives arising is expected to increase. However, the consistently positive effects of tai chi—seen in small to moderate DNA methylation change over time—for all six marks showing a significant difference between the two cohorts lend support that the changes observed reflect a true, positive biological benefit of the tai chi practice. The nonrandomised design of this study also makes it more difficult to control for the effects of confounders. These effects have been minimised by the well-matched general characteristics of the two cohorts and by our statistical model including potential confounders that may be related to DNA methylation. However, further randomised studies would be useful. This study investigated loci-specific CpGs changes. Further work will benefit from looking at a genome-wide DNA methylation profile of tai chi. In our study, we chose “beginners” versus “3 or more years” to try to ensure that we have two sufficiently distinct groups—one with significant tai chi experience and one with virtually no experience. More precise determination of tai chi experience examining the effects of intensity and duration of tai chi practice on epigenetic outcomes would be of interest. In addition, it would be informative to investigate other parameters such as tissue types, younger individuals, the males, the effects of short-term practice, and the styles of tai chi and methods of teaching used.

## 5. Conclusion

This work has provided preliminary evidence that tai chi practice may be associated with measurable beneficial epigenetic changes. The ability to measure such changes at the molecular level opens up the possibility of designing new and objective approaches that can be used to delineate the biological mechanisms and the health and therapeutic efficacies of tai chi. Such approaches may also benefit the study of other popularly used forms of complementary and alternative medicine interventions such as acupuncture, herbal medicine, meditation, qigong, Reiki, Shiatsu and yoga.

## Figures and Tables

**Table 1:** Characteristics of the female participants*.

Characteristics	Tai Chi (*N* = 237) %^†^	Control (*N* = 263) %^†^	Difference (*P* value)
Age (year) (mean ± SD)	64.6 ± 10.4	62.6 ± 10.9	0.71
Education level			0.59
Secondary	32	27	
Tertiary	31	31	
Degree (university)	35	40	
Other	3	2	
Have children	81	80	0.66
Occupation			0.14
Employed	36	42	
Homemaker	9	10	
Retired	54	45	
Other	1	3	
Born in Australia	71	70	0.83
Ethnic background			0.66
European^‡^	90	89	
Asian^‡^	5	6	
Other	5	5	
Regular non-tai chi exercise	86	85	0.99
Do yoga or meditation	3	4	0.37
Smoke			0.84
Never smoked	82	76	
Smoked in the past	15	19	
Still smoking	3	5	
Medical conditions			
Type 2 diabetes	3	6	0.55
Hypertension	32	25	0.06
Cardiovascular disease	4	7	0.16
Body mass index^§^			0.61
Underweight	14	12	
Normal	50	45	
Overweight	23	23	
Obese	13	20	
Situational anxiety^¶^	32.8 ± 9.8	35.3 ± 11.4	0.04^‡‡∗∗^
Perceived stress^*||*^	4.5 ± 2.6	4.8 ± 2.9	0.28^‡‡^
Degree of stress^††^	7.2 ± 2.0	6.9 ± 2.1	0.32^‡‡^

*Self-report data from questionnaire.

^†^All values (except age) are given as a percentage of the total number of participants in the cohort.

^‡^“European” refers to both Northern and Southern European origins. “Asian” refers to Chinese and Indian origins.

^§^Body mass index (BMI) is weight in kilograms divided by the square of height in meters. Underweight (BMI < 18.5); normal weight (BMI 18.5–24.9); overweight (BMI 25–29.9); obese (BMI ≥ 30).

^¶^Participants were asked to describe how they felt presently at the moment of answering the questionnaire using the state subscale of the State-Trait Anxiety Inventory (STAI) (mean ± SD), where the range of scores for the STAI is 20–80, 80 being the highest level of STAI.

^*||*^Participants were asked to describe their feelings and thoughts during the last month at the time of answering the questionnaire using the Perceived Stress Scale (mean ± SD) on a scale of 0–16, 16 being the highest level of PSS.

^††^Participants were asked if they had experienced significant stressful life events in the past 12 months, for example, the death of someone close, diagnosis of a serious health condition in the participant or someone close. Values (mean ± SD) denote the degree of stress on a scale of 1–10, 10 being the highest level of stress.

**A significant difference between the tai chi and control cohorts.

^‡‡^ *t*-test was performed for these continuous data instead of the Chi-squared tests used for the other categorical data.

**Table 2:** Reason(s) for practising tai chi*.

Reasons	Tai chi cohort %^†^ (*N* = 237)
To help with a specific health problem	26
For relaxation	63
For physical wellbeing	84
For mental wellbeing	65
To feel good	62
Other	8

*As reported in the questionnaire by the tai chi practitioners, who were asked to tick as many categories as applicable.

^†^Values are given as a percentage of the total number of participants in the tai chi cohort.

**Table 3:** Genetic loci and primer sequences used for DNA methylation analysis.

Genetic locus	Primer	Primer sequence	Product size (bp)	Physical location	No. of CpGs
Rad 50	Forward	GGAGTTTTAGGATTAAGTTTTGGGA	467	Chr5: 131920312–131920779	18
	Reverse	CTACCAAAAAATCCTACCAACCAA			
17p (subtel)	Forward	AGTGAGGAAGAGGTTTTTGGTTTAT	229	Chr17: 2774–3003	8
	Reverse	ACTAAACCCCTACAACTAAATCCCA			
G6PD	Forward	GGAAAAGTTGAGGTATGGAGTAGGT	378	ChrX: 153427381–153427759	8
	Reverse	TAAAACCCAAAACCAACAATTTCTA			
Xp13 (subtel)	Forward	TGGGTTTAGGTGTTATTGAGGATAG	309	ChrX: 34124–34433	2
	Reverse	CCCAATAAAAACCCTAAAAATTACA			
Ercc1	Forward	TGTGTGTTTAGAATTTTGGTAGGGT	413	Chr19: 50619182–50619595	10
	Reverse	CTACTCAAATATCCCTTCCTCCAA			
Esr 1	Forward	TGGTAGGTTGTATTTTTTTGATGGT	265	Chr6: 152169890–152170155	4
	Reverse	ATTTATCTCTCTTTCTATTTAATTCCCCC			
WRN	Forward	TTTGTTTTTAGGATATTGTGAGGATG	444	Chr8: 31009321–31009765	10
	Reverse	CAAAAATAAACTCTATCCCCAAACC			

**Table 4:** CpG marks showing a significant methylation difference between tai chi and control cohorts across age.

CpG mark	Chromosomal location of CpG	Mean methylation difference (± SD)	*P* value
Rad50_2	Chr5: 131920753	0.081 ± 0.033	0.022
17P_7	Chr17: 2913 (subtelomeric)	0.019 ± 0.008	0.019
G6PD_6	ChrX: 153427632	0.027 ± 0.010	0.013
G6PD_7	ChrX: 153427612	0.038 ± 0.015	0.032
Rad50_10	Chr5: 131920606	−0.025 ± 0.011	0.008
Xp13_1	ChrX: 34302 (subtelomeric)	−0.106 ± 0.032	0.016
